# Retraction Note: Down-regulation of microRNA-342-5p or Up-regulation of Wnt3a Inhibits Angiogenesis and Maintains Atherosclerotic Plaque Stability in Atherosclerosis Mice

**DOI:** 10.1186/s11671-023-03832-6

**Published:** 2023-03-20

**Authors:** Haixia Sun, Jinhua Feng, Yan Ma, Ding Cai, Yulu Luo, Qinggong Wang, Fang Li, Mingyue Zhang, Quanzhong Hu

**Affiliations:** 1 Department of Cardiac Ultrasound, Qinghai Provincial People’s Hospital, Xining, 810007 Qinghai Province China; 2 Department of General Practitioner, Qinghai Provincial People’s Hospital, Xining, 810007 Qinghai China; 3 Department of Cardiac Ultrasound, Haixi People’s Hospital, Delingha, 817099 Qinghai China; 4 Department of Neurology, Qinghai Provincial People’s Hospital, No. 2 Gonghe Road, East District, Xining, 810007 Qinghai Province China

**Retraction note: Nanoscale Research Letters (2021) 16:165** https://doi.org/10.1186/s11671-021-03608-w

The Editors in Chief have retracted this article. After publication, concerns were raised concerning the authors' contributions and the integrity of the article. The authors did not respond to requests for further clarification and did not supply raw data or the ethics permit. The Editors, therefore, have lost confidence in the integrity of the article's findings. The authors did not respond to any correspondence from the Editor about this retraction.

